# Chinese Private Preschool Teachers’ Teaching Readiness and Teacher–Child Relationships: The Chain Mediation Effects of Motivation to Teach and Self-Efficacy

**DOI:** 10.3390/bs13110900

**Published:** 2023-10-31

**Authors:** Hui Li, Wei Chen, Huihua He, Wenwei Luo

**Affiliations:** 1 Shanghai Institution of Early Childhood Education, Shanghai Normal University, Shanghai 200234, China; 1000529798@smail.shnu.edu.cn (H.L.); wenweiluo@shnu.edu.cn (W.L.); 2 Shanghai Business School, College of International Education; Shanghai 200235, China; chenwei@sbs.edu.cn

**Keywords:** private preschool, teaching readiness, teacher–child relationship, motivation to teach, self-efficacy

## Abstract

The teacher–child relationship is a key element in measuring the quality of childcare institutions and is essential to the current and future physical and mental developmental outcomes of children. The purpose of this study is to investigate the role of private preschool teachers’ readiness to teach in influencing the teacher–child relationship, and to explore the mechanisms by which teachers’ motivation and self-efficacy mediate their readiness to teach and the teacher–child relationship. Online questionnaires were administered to 289 early-childhood teachers in Shanghai, China. The findings of the study indicated a significant and positive correlation between early-childhood teachers’ readiness to teach and the quality of the teacher–child relationship. In addition, early-childhood teachers’ internal motivation to teach and self-efficacy mediated their readiness to teach and the teacher–child relationship, respectively. However, teachers’ external motivation did not mediate the effect of teaching readiness on the quality of the teacher–child relationship. Moreover, teachers’ motivation to teach (encompassing both internal and external factors) and their self-efficacy played chained roles in mediating the relationships between teaching readiness and the teacher–child relationship. This study highlights the significant roles of teaching readiness and instructional motivation, along with self-efficacy, in cultivating positive teacher–child relationships within early-childhood education settings.

## 1. Introduction

High-quality teacher–child relationships create a protective and supportive environment that serves as a critical indicator of the quality of kindergarten education and fosters children’s development [1]. The National Association for the Education of Young Children [2] has identified positive teacher–child relationships as a key accreditation standard for early-childhood education programs. Prior research has extensively demonstrated the pivotal role of the teacher–child relationship in children’s academic, emotional, and social development [3,4,5,6]. For example, Hu et al. (2017) confirmed that high-quality teacher–child interactions significantly and positively predicted the development of children’s cognitive skills [5]. Paes et al. (2023) found that teacher–child conflict significantly predicted poorer social skills in young children, particularly leading to reduced self-confidence and engagement [4]. In addition, teacher–child relationships also play a significant role in fostering teachers’ engagement. A positive teacher–child relationship can imbue a teacher’s work with meaning and value, bolstering their professional identity and fostering a greater willingness to engage in ongoing professional development and daily teaching practices [7,8]. Therefore, investigating the mechanisms that shape high-quality teacher–child relationships can effectively inform teachers’ preparedness and professional development [9].

The quality of teacher–child relationships is influenced by both the teacher’s characteristics and the child’s characteristics [10,11]. However, teachers play a dominant role in the teacher–child relationship [12]. Evidence has demonstrated that the teacher’s characteristics exert a more significant influence on the quality of the teacher–child relationship compared to the child’s characteristics [13]. Building upon previous research findings, the present study intends to employ a variable-centered approach to examine the roles played by three teacher characteristics—readiness to teach, motivation to teach, and self-efficacy—in shaping the teacher–child relationship.

### 1.1. Teaching Readiness (TR) and the Teacher–Child Relationship (TCR)

In 1990, Housego defined teachers’ teaching readiness (TR) as the degree to which an individual teacher is psychologically prepared or anticipates their professional qualities and competencies [14]. Prior research has indicated that TR originates from pre-service teacher education programs and can also be attained from in-service training [15]. Several scholars have also highlighted that teachers’ readiness to teach is influenced by their self- assessment, which may not accurately reflect their educational and instructional competencies in authentic educational settings. However, this self-appraisal process also contributes to the cultivation of teachers’ confidence in education and teaching, as well as their professional development [16]. Therefore, TR forms the foundation of teachers’ professional development and serves as the root of their educational beliefs about teachers, children, and teaching throughout their careers. Prior research has shown that teachers’ degree of professional development is critical to the quality of the teacher–child relationship [17]. Nevertheless, limited research has directly investigated the impact of teachers’ readiness to teach on teacher–child relationships. Some studies have found that factors such as the complexity of practitioners’ explanations and their level of qualification are critical proximal variables that affect the quality of teacher–child interactions [18,19]. The level of qualification of teacher practitioners is, to some extent, an objective indicator of the quality of teacher preparation, indicating the level of teachers’ mastery of the theoretical knowledge related to child development. Drawing on this knowledge, teachers can offer comprehensive explanations and develop a deeper understanding of children’s developmental needs and behaviors [20], enabling them to foster more positive teacher–child interactions. Based on these research findings, Hypothesis 1 is as follows:

**H1.**  *Teaching readiness positively predicts the teacher–child relationship.*

### 1.2. Motivation to Teach (MT) as a Mediator

Motivation refers to the innate energy and drive that propel an individual to naturally engage in a task. It indicates both the underlying reasons prompting an individual’s engagement in a task and the degree of effort that they are willing to invest in pursuing it [21,22]. Accordingly, teacher’s MT could be defined as the reasons why an individual chooses to teach and sustains the intrinsic value to engage in the teaching profession, as well as the commitment and level of effort that they dedicate to teaching [23].

The Factors Influencing Teaching Choice (FIT-Choice) is an internationally recognized theoretical framework for evaluating teachers’ MT [24,25] and has been widely used in research on teachers’ MT in various countries across the world [23,26]. This theory suggests that the main factors that construct MT include prior teaching and learning experiences, perceived teaching competence, intrinsic professional values, and personal and social utility values [27,28]. A teacher’s MT is largely a centralized reflection of their teaching experience, their teaching skills and techniques, and their beliefs about the value of education. Accordingly, there is a close relationship between teachers’ readiness to teach and their motivation to teach [29].

Moreover, teachers’ MT is an important factor in the quality of their work [30] and is critical to the growth of students and the wellbeing of individual teachers. MT not only affects their own professional goals, teaching behaviors, and job satisfaction, but also significantly impacts students’ motivation and learning [31]. As Zou et al. (2023) found in their research, teachers’ motivation to teach shapes the ways in which teachers motivate their students, which, in turn, affect the quality of the teacher–student relationship [32]. Drawing from these findings, we believe that there is a close correlation between teachers’ motivation to teach and the teacher–child relationship. Building upon the above findings, we propose research Hypothesis 2:

**H2.**  *MT mediates the effects of TR on the teacher–child relationship.*

### 1.3. Self-Efficacy (SE) as a Mediator

Self-efficacy beliefs are generally characterized as the belief in one’s ability to activate and maintain the course of action needed to achieve desired outcomes [33]. Past research has demonstrated that teachers’ pre-service education and preparation are important factors in the formation of SE [34,35]. For example, a large empirical study of two groups of U.S. teachers—both early intervention teachers and preschool special education teachers—found that teachers’ sense of preparedness was the best predictor of SE [36]. As Lewis et al. (1999) argued, a teacher who has a more adequate self-perceived readiness to teach is likely to be a highly qualified teacher, as this indicates that they have gained the expertise and skills that are essential for effective teaching [37]. Thus, teachers’ higher readiness to teach indicates that they are more likely to have the competence to teach in specific domains, which, in turn, will generate higher levels of SE.

In addition, prior empirical studies have found a strong link between teachers’ SE and teacher–student relationships [38,39,40]. For instance, Summers et al. (2017) discovered that teachers’ SE beliefs significantly enhanced intimacy and reduced conflict in teacher–student relationships [39]. Moyano et al. (2023) also found that teachers with higher SE were more inclusive of student behavior and demonstrated more positive work engagement [40]. Teachers who are confident in their teaching and classroom management skills respond more effectively and engage emotionally with their students’ needs. In addition, they can allocate more energy to fostering supportive and caring relationships with their students [41]. In conclusion, the higher a teacher’s level of SE, the more likely it is that positive relationships between teachers and young children will be enhanced. Therefore, we propose the following:

**H3.**  *Teachers’ SE beliefs mediate the relationship between TR and the teacher–child relationship.*

### 1.4. The Chain Mediation Effect of MT and SE

Previous studies have indicated that teachers’ perceived teaching abilities or self-reported TR and personal and social utility values may play significant roles in shaping individuals’ teaching choices and MT [25]. Teachers’ SE could also serve as a critical indicator or outcome of TR [15]. The feelings of proficiency in teaching and engaging with children are crucial in the development of teachers’ SE beliefs. In addition, many studies have revealed that teachers who rate themselves as being ill-prepared to teach may have negative experiences in the classroom, resulting in low MT and the inability to construct high levels of SE [16].

**Figure 1:**
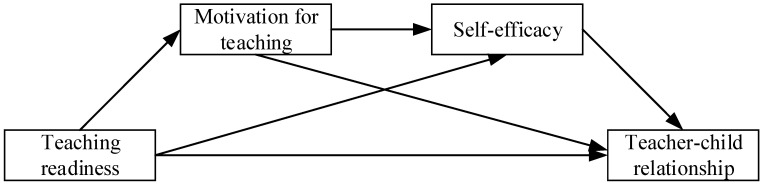
Hypothetical mediation model.

Moreover, moderate associations between teachers’ MT and SE have been identified from the existing literature [42]. For example, a study conducted on trainee teachers confirmed that individuals’ motivation to teach highly influences teachers’ future self-efficacy [43]. Bilim (2014) also demonstrated that teachers’ motivation to teach was strongly related to self-efficacy, and that teachers with high intrinsic motivation have higher self-efficacy [42]. An escalated intensity of teachers’ motivation to teach indicates that they are more passionate and motivated about the teaching profession and, thus, are more willing to put effort into their work, which then enhances their self-efficacy. Furthermore, studies exploring the impact factors of teachers’ professional development (PD) suggest that SE mediates the effects of MT on teachers’ commitment to participate in training activities. MT could inspire teachers’ desire for PD and their willingness to denote personal time for professional meetings. Based on these studies, the present study hypothesizes the folloeing:

**H4.**  *MT and SE play a chain-mediating role in the relationship between TR and the teacher–child relationship (Figure 1).*

## 2. Methods

The data for this study were derived from a large-scale study on the professional development of private preschool teachers in Shanghai, China. This municipal-level research involved all teachers from all private preschools, with stratified sampling to establish the final sample. The purpose of the study was to understand the experiences of individuals working as private preschool teachers, as well as to identity the specific requirements and challenges that they encounter in the realms of teaching and professional development. Our research employed a questionnaire approach, mainly derived from the relevant parts of the OECD’s TALIS questionnaire. This instrument was subsequently modified by experts in early-childhood education in Shanghai, China to suit the specific requirements of the preschool domain.

### 2.1. Participants

Stratified random sampling was conducted to select preschool teachers from various kindergartens in Shanghai, China as the research object. Using administrative districts as a stratification criterion, the research team sampled two private kindergartens from each of the five administrative districts of Shanghai, including the Xuhui, Putuo, Minhang, Pudong New Area, and Jinshan districts. Furthermore, all teachers in each kindergarten were invited to participate in this study. Finally, online questionnaires were distributed to 10 kindergartens from the five administrative districts in Shanghai through Wenjuanxing (www.wjx.cn, accessed on 5 June 2023) (an online survey platform in China). A total of 289 valid questionnaires were collected. The participants ranged in age from 19 to 62 years old, with a mean age of 32.13 years (SD = 8.03). The participants’ years of teaching experience ranged from 0 to 30 years, with a mean of 6.07 years and a standard deviation of 5.56 years. Among them, 284 were female, accounting for 98.30%, while 196 teachers held a bachelor’s degree or above, accounting for 67.90%. This study was reviewed and approved by the Academic Ethics Committee of Shanghai Normal University, and all participants were informed of the purpose and procedures of the research.

### 2.2. Measures

#### 2.2.1. Teacher–Child Relationship

This study drew on the teacher–student relationship scale from the Teaching and Learning International Survey [44] developed by the Organization for Economic Co-operation and Development (OECD) to measure teacher–child relationships. The original scale consisted of 5 items and was scored on a 4-point Likert-type scale from 1 (strongly disagree) to 4 (strongly agree). Higher total scores indicate better quality of teacher–child relationships. We revised the word “students” to “children” in the items of the original scale to meet the terminological conventions of preschool education. For example, we amended the item “Teachers and students usually get on well with each other” in the original scale to “Teachers and children usually get on well with each other”. In addition, the item “Teachers can rely on each other” in the original scale was deleted in this study because it does not measure the quality of teacher–child relationships per se. Eventually, the questionnaire was revised to retain 4 items. In this study, the Cronbach’s alpha coefficient for the teacher–child relationship was 0.94. Meanwhile, the composite reliability (CR) and the average variance extracted (AVE) of the scale were 0.94 (>0.70) and 0.79 (>0.50), respectively. These indicators suggest that the scale has acceptable reliability and validity.

#### 2.2.2. Teachers’ Teaching Readiness

The Teacher Readiness for Teaching Questionnaire was adapted from the Teacher Readiness Questionnaire in the TALIS 2018 Teacher Questionnaire. Based on the original scale, the research team revised the items of the original scale in a culturally adapted manner, taking into account the specificity of preschool education and the characteristics of preschool education in China. For instance, we amended the original item “Content of some or all subject(s) I teach” to “Knowledge and core experience in various fields I teach”. The adapted questionnaire consisted of 11 items, and all items were scored on a 4-point Likert-type scale from 1 to 4 (1 = not at all, 2 = somewhat, 3 = well, 4 = very well). The total score for teachers’ teaching readiness was calculated by adding all the scores for individual items. Higher scores indicate higher teaching readiness possessed by the teachers. The Cronbach’s alpha coefficient of the scale was 0.94 in this study. Confirmatory factor analysis (CFA) revealed good model fit indices for this scale: *χ^2^*/*df* = 2.88, *p* < 0.001, root-mean-square error of approximation (RMSEA) = 0.08, comparative fit index (CFI) = 0.98, Tucker–Lewis index (TLI) = 0.97, incremental fit index (IFI) = 0.95, and goodness of fit index (GFI) = 0.94.

#### 2.2.3. Teachers’ Motivation to Teach

This study drew on the “Motivation for Teaching Questionnaire” from the TALIS 2018 Teacher Questionnaire to measure the motivation of teachers. This questionnaire consists of two dimensions: external motivation to teach (4 items), and internal motivation to teach (3 items). The research team revised the original items appropriately based on the original scale, incorporating preschool terminology conventions and the Chinese cultural background. External motivation reflects the extent to which teachers recognize the external incentives (e.g., income, vacations, social prestige, career path, etc.) associated with their profession. Sample item: “Good wages as a kindergarten teacher”. Internal motivation to teach reflects the extent to which teachers recognize that their profession satisfies their intrinsic personal interests. Sample items: “I love children”, “I have passion for preschool education”, and “Being a kindergarten teacher enables me to realize the value of my life”. Items were scored on a four-point Likert scale from 1 to 4 as “Not important at all” or “of high importance”. The higher the score, the higher the teacher’s motivational propensity for teaching. For this study, the Cronbach’s alpha coefficient for the whole scale was 0.86. The internal consistency coefficients of external motivation for teaching and internal motivation for teaching were 0.79 and 0.83, respectively. The CFA indicated that the scale had a good fit, with *χ^2^*/*df* = 2.31, *p* < 0.001, RMSEA = 0.07, CFI = 0.99, TLI = 0.97, IFI = 0.99, and GFI = 0.98.

#### 2.2.4. The Self-Efficacy of Preschool Teachers

The Teachers’ Sense of Efficacy Scale (TSES), compiled by Tschannen-Moran et al., was used to measure the levels of teachers’ self-efficacy in this study [45]. The scale consists of three dimensions: efficacy for classroom management (4 items, e.g., “How much can you do to control disruptive behavior in the classroom”), efficacy for instructional strategies (4 items, e.g., “To what extent can you craft good questions for your students”), and efficacy for student engagement. Given the contents of the scale and the actual work of preschool teachers, only dimensions of efficacy for classroom management and efficacy for instructional strategies were selected for this study. The items were scored on a 5-point Likert-type scale, ranging from “Very little” (scored 1 point) to “Very much” (scored 5 points), with higher scores indicating a higher degree of self-efficacy. In this study, the Cronbach’s alpha coefficient for the whole scale was 0.96. The Cronbach’s alpha coefficients for classroom management and instructional strategies were 0.95 and 0.94, respectively. The results of CFA verified the structure of the scale as a model that was adequately fitted to the data, with *χ^2^*/*df* = 2.81, *p* < 0.001, RMSEA = 0.08, CFI = 0.99, TLI = 0.98, IFI = 0.99, and GFI = 0.96.

### 2.3. Data Analysis

The data collection and analysis in this study were conducted using SPSS 26.0 software. Descriptive data on the main variables of the teacher–child relationship, teachers’ teaching readiness, self-efficacy, and motivation for teaching were reported using means and standard deviations. The correlation between these variables was analyzed using Pearson’s product-moment correlation method. Harman’s single-factor test was conducted to test for possible common methodological bias in this study [46]. The mediating effects of teachers’ motivation to teach and self-efficacy on the relationship between teachers’ teaching readiness and the teacher–child relationship were examined using model 6 of the PROCESS plug-in for the SPSS macro program developed by Hayes [47]. To examine the significance of the regression coefficients of the parametric test, the bias-corrected bootstrap method was used in this study. Meanwhile, 5000 bootstrap samples with retraction were drawn to estimate a 95% confidence interval of the mediating-effect value in the model.

## 3. Results

### 3.1. Common Method Bias

Since the variables of teaching readiness, teacher–child relationship, motivation to teach, and self-efficacy were all measured by subjects’ self-reports in this study, the Harman single-factor test was employed to test for common method bias (that is, perform the unrotated principal component analysis on all variables simultaneously). According to the results, there were five factors with eigenvalues greater than one, and the variance explained by the first of these factors was 35.29%, which was less than the critical value of 40%. The results indicated that there was no significant common method bias in this study, and further data analysis could be carried out.

### 3.2. Preliminary Analysis

The mean, standard deviation, and correlation matrix for each variable are shown in Table 1. The means and standard deviations (M ± SD) of the teaching readiness, teacher–child relationship, external motivation to teach, internal motivation to teach, and self-efficacy were 3.13 *±* 0.57, 3.59 ± 0.56, 3.60 ± 0.55, 3.80 ± 0.38, and 3.99 ± 0.63, respectively.

In addition, the correlation between teaching readiness, teacher–child relationship, external motivation to teach, internal motivation to teach, and self-efficacy was analyzed using bivariate correlation. The results showed that the teacher–child relationship was significantly positively correlated with teaching readiness (*r* = 0.27, *p* < 0.01), external motivation to teach (*r* = 0.17, *p* < 0.01), internal motivation to teach (*r* = 0.26, *p* < 0.01), and self-efficacy (*r* = 0.31, *p* < 0.01). Teachers’ teaching readiness was significantly positively correlated with external motivation to teach (*r* = 0.42, *p* < 0.01), internal motivation to teach (*r* = 0.35, *p* < 0.01), and self-efficacy (*r* = 0.26, *p* < 0.01). Teachers’ self-efficacy was significantly positively correlated with external motivation to teach (*r* = 0.25, *p* < 0.01) and internal motivation to teach (*r* = 0.24, *p* < 0.01). Furthermore, a significant positive correlation was found between teachers’ external motivation to teach and internal motivation to teach (*r* = 0.64, *p* < 0.01).

### 3.3. Test of the Mediating Effects of Motivation to Teach and Self-Efficacy

The bias-corrected bootstrap method was used to verify the mediating role of motivation to teach and self-efficacy in the relationship between teaching readiness and the teacher–child relationship. The results of the regression analysis showed that teachers’ teaching readiness significantly and positively predicted their teacher–child relationships (*β* = 0.27, *p* < 0.001) when no mediating variables were included. Since the two components of teachers’ motivation for teaching as a mediating variable, i.e., internal and external motivation, have different effects on the teacher–child relationship, they were tested separately.

#### 3.3.1. Roles of Teachers’ Internal Motivation to Teach and Self-Efficacy

After including the mediating variables of internal motivation to teach and self-efficacy (Table 2, Model 1), the direct predictive effect of teachers’ teaching readiness on the teacher–child relationship was significant (*β* = 0.16, *p* < 0.01), as well as on internal motivation to teach (*β* = 0.35, *p* < 0.001) and on teachers’ self-efficacy (*β* = 0.20, *p* < 0.001). Meanwhile, the teachers’ teaching readiness was found to affect their self-efficacy significantly and positively (*β* = 0.17, *p* < 0.01), as well as their teacher–child relationships (*β* = 0.15, *p* < 0.05), while teachers’ self-efficacy was also found to have a significant effect on teacher–child relationships (*β* = 0.23, *p* < 0.001).

**Figure 2:**
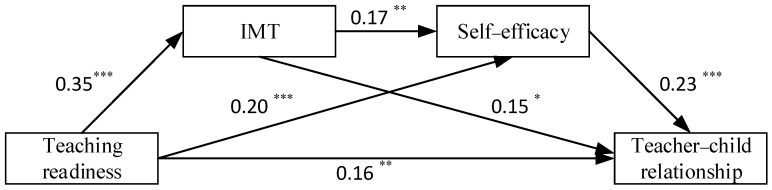
Mediation model examining the indirect relationship between teaching readiness and teacher–child relationship through internal motivation to teach and self-efficacy. Note: * *p* < 0.05; ** *p* < 0.01; *** *p* < 0.001. IMT = internal motivation to teach.

We generated 5000 bootstrap samples with a confidence level of 95% to estimate the mediating effect value. The results revealed that the mediating role of teachers’ internal motivation to teach and self-efficacy was significant in readiness to teach and teacher–child relationships. As shown in Table 3 and Figure 2, the size of this total indirect effect was 0.110, 95% CI = [0.058, 0.172], and the ratio of the mediating effect to the total effect (0.271) was 40.59%. Specifically, the value of the indirect effect of a single mediated path for teachers’ internal motivation was 0.051 (95% CI = [0.014, 0.090], ratio to total effect = 18.82%), the value of the indirect effect of a single mediated path for self-efficacy was 0.046 (95% CI = [0.013, 0.088], ratio to total effect = 16.97%), and the value of the indirect effect of a chained mediated path for teachers’ internal motivation and self-efficacy was 0.013 (95% CI = [0.004, 0.028], ratio to total effect = 4.80%). The 95% confidence intervals for the indirect effects of all three paths do not contain zero; therefore, the mediating effect of all three paths is significant.

#### 3.3.2. Roles of Teachers’ External Motivation to Teach and Self-Efficacy

Furthermore, as shown in Model 2 in Table 2, the inclusion of two mediating variables—external motivation to teach and self-efficacy—revealed that teachers’ teaching readiness still significantly and positively influenced external motivation to teach (*β* = 0.42, *p* < 0.001), self-efficacy (*β* = 0.19, *p* < 0.01), and teacher–child relationships (*β* = 0.20, *p* < 0.01). In addition, teachers’ self-efficacy can significantly positively predict teacher–child relationships (*β* = 0.25, *p* < 0.001), and self-efficacy (*β* = 0.17, *p* < 0.01) can also be positively predicted by external motivation to teach. However, teachers’ external motivation to teach could not predict teacher–child relationships (*β* = 0.03, *p* > 0.05).

**Figure 3:**
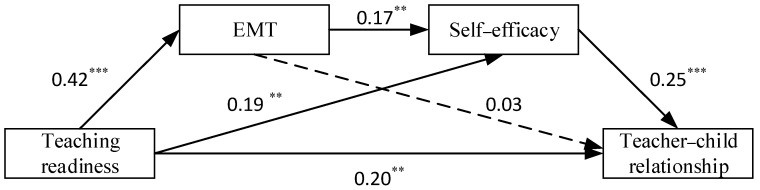
Mediation model examining the indirect relationship between teaching readiness and teacher–child relationships through external motivation to teach and self-efficacy. Note: ** *p* < 0.01; *** *p* < 0.001. EMT = external motivation to teach.

The results of the mediation effects analysis (Table 3 and Figure 3) showed that the size of this total indirect effect was 0.075, 95% CI = [0.013, 0.144], and the ratio of the mediating effect to the total effect (0.271) was 27.68%. Specifically, the value of the indirect effect of a single mediated path for teachers’ external motivation to teach was 0.011 (95% CI = [−0.041, 0.058], ratio to total effect = 4.06%), the value of the indirect effect of a single mediated path for self-efficacy was 0.047 (95% CI = [0.013, 0.094], ratio to total effect = 17.34%), and the value of the indirect effect of a chained mediated path for teachers’ external motivation to teach and self-efficacy was 0.017 (95% CI = [0.006, 0.034], ratio to total effect = 6.27%). However, since the 95% confidence interval for the indirect effect of the single mediated path of teachers’ external motivation to teach contains zero, it suggests that the mediating effect of this path is not significant.

In summary, our study concluded that teachers’ readiness to teach is an essential antecedent to increase the quality of the teacher–child relationship, so H1 was supported. Teachers’ internal motivation to teach imperfectly mediated the relationship between teachers’ readiness to teach and the teacher–child relationship, so H2 was supported, but it should be noted that the mediating effect of teachers’ external motivation to teach was not significant. In addition, H3 was also supported, as this research found that teachers’ self-efficacy also played an incomplete mediating role in the relationship between teachers’ readiness to teach and the teacher–child relationship. Finally, H4 was also supported by this study, as it proved that teachers’ motivation to teach and self-efficacy play a significant role as chain mediators in teachers’ readiness to teach and teacher–child relationships.

## 4. Discussion

In summary, the findings of this study indicate that TR plays a direct role in influencing teacher–child relationships. In addition, an indirect impact on teacher–child relationships through mediators of MT and SE was also found. These results provide a new theoretical perspective about improving the quality of teacher–child relationships and enhancing TR and SE.

### 4.1. The Relationship between TR and Teacher–Child Relationships

This study found that teachers’ readiness to teach significantly and positively predicted teacher–child relationships. To the best of our knowledge, little research has explored the direct relationship between these two variables. Therefore, the present study fills a research gap and can provide a new theoretical horizon for research related to the influence of post-service teacher characteristics on the quality of teacher–child relationships. The knowledge and beliefs that teachers possess play a profound role in impacting their teaching practices [48,49]. The comprehensive knowledge and beliefs about child development and teaching that early-childhood teachers uphold affect the ways in which they interact with young children and, thus, carry on affecting the quality of the teacher–child relationship [49,50,51,52]. Accordingly, a higher level of teacher preparedness means that teachers have good knowledge and beliefs about child development and teaching and are more inclined to interact with children through appropriate pedagogical practices. Therefore, this study suggests that policymakers, teacher educators, and kindergarten administrators need to pay attention to the quality of pre-service teacher education and post-service teacher professional development programs to effectively enhance teachers’ readiness to teach.

### 4.2. Mediating Role of Teachers’ Motivation to Teach and Self-Efficacy

Notably, we found that the participants’ internal MT had a higher mean value than their external MT. This suggests that the Chinese teachers’ motivation to teach is predominantly internal—a finding that is consistent with several previous China-based studies [53,54]. Teachers’ motivational characteristics may be closely related to the social and cultural background of China, as China possesses a cultural tradition that honors teachers, practices collectivist values, and holds the dedication of teachers in higher esteem.

Further mediating-effects tests showed that teachers’ internal motivation to teach mediated the effects of their readiness to teach and the teacher–child relationship, but the mediating effect of teachers’ external motivation to teach was not of considerable significance. This study confirmed that teachers’ internal motivation to teach highly predicted the quality of the teacher–child relationship, which also supports previous research [32,55]. According to the self-determination theory, the internal motivation of teachers to teach is one of the most autonomous forms of motivation [56], so that teachers engage in the teaching profession with the aim of deriving pleasure and satisfaction from the educational activity itself. The stronger the internal motivation of a teacher, the more that teacher likes children, enjoys teaching, and is happy to invest and dedicate themself to preschool education [57]. In addition, teachers’ internal motivation is also closely related to best teaching practices [58]. Therefore, teachers’ internal motivation to teach will inspire teachers to engage in teacher–child interactions with more positive affective attitudes and more effective teaching behavior strategies, which, in turn, will enhance the quality of the teacher–child relationship. However, this study found that teachers’ external motivation to teach did not significantly predict the quality of teacher–child relationships. This may be because the positive value of teachers’ external motivation needs to be mediated or moderated by other factors, such as job satisfaction, sense of efficacy, etc.

In addition, teachers’ self-efficacy plays a partial mediating role between teachers’ readiness to teach and the teacher–child relationship. The findings of this study were consistent with previous research, showing that teachers’ readiness to teach affects the development of their self-efficacy [36]. Echoing Piaget’s theory of cognitive development, a teacher’s readiness to teach can be understood as a personal schema; the higher the readiness to teach, the stronger and more complete the schema [59]. Therefore, the higher the teacher’s readiness, the more able they are to use existing schemas to solve problems in the work situation through assimilation, and consequently, the greater the self-efficacy. Meanwhile, consistent with previous research [38,60], this study also confirmed that teachers’ self-efficacy significantly and positively predicts teacher–student relationships, which provides empirical evidence to support the important role of teacher’s self-efficacy in teacher–child relationships. Furthermore, Yin et al. (2022) reported that most of the research on the impact of self-efficacy on teacher–student relationships has not considered the sources of teachers’ self-efficacy beliefs [60]. In contrast, the present study pushes the boundaries of theoretical research by proposing a mechanism for the mediating role of self-efficacy in teachers’ readiness to teach and the teacher–child relationship, and by elucidating the positive role of readiness to teach in promoting teachers’ self-efficacy beliefs.

### 4.3. The Chain Mediating Effect of Teachers’ MT and SE

The present study further found a serial mediation role for teachers’ motivation to teach (internal and external motivation) and self-efficacy in the relationship between teachers’ readiness to teach and the teacher–child relationship. Adequate readiness to teach enhances teachers’ level of motivation to teach, which, in turn, enhances their sense of self-efficacy, thus constructing good-quality teacher–child relationships. Previous studies have mainly explored the negative effects of teachers’ external motivation to teach, e.g., it enhances burnout and reduces job happiness and professional commitment [57]. However, this study confirms the positive aspects of teachers’ external motivation, i.e., teachers’ external motivation to teach can influence teacher–student relationships through teachers’ self-efficacy in a positive way. However, while internal motivation is preferred, this study suggests that external motivation, though not always as sustainable as intrinsic motivation, should be reconsidered for its beneficial role, especially in specific situations. Given that teachers’ external motivation is cultivated and effectively utilized, it can also play a positive functional role in early childhood workplaces.

## 5. Conclusions, Limitations, and Implications

In conclusion, enhancing the quality of teacher–child relationships is a core initiative to ensure the quality of teaching and learning in early-childhood education settings, and teachers’ characteristic factors, as proximal factors affecting teacher–child relationships, deserve special attention. This study examined the relationships between proximal factors such as teachers’ readiness to teach, teachers’ motivation and self-efficacy, and the teacher–child relationship, and further verified the mechanisms by which these proximal factors contribute to the teacher–child relationship.

However, there are several notable limitations to the present study. First, due to the specific nature of the early-childhood teacher population, there was a significant gender imbalance in the participants recruited for this study (i.e., only five male teachers). This may affect the science and generalizability of the findings. Therefore, future studies should try to ensure equitable representation of both genders among the participants. Second, this study only focused on the role of teachers’ characteristics in influencing teacher–child relationships and did not take account the effects of child factors, organizational environment factors, and other factors on teacher–child relationships. Therefore, future research could employ hierarchical linear modeling (HLM) [61] to test the interaction effects of teacher factors, child factors, and organizational environment factors on teacher–child relationships. In addition, the mediation model constructed in this study also could be further enriched and optimized. For example, teachers’ motivation, as a mediator, may also be influenced by multiple factors, such as the physical environment of the kindergarten, the leadership of the director, coworker relationships, job satisfaction, and social culture. Therefore, future research could further explore the mediating or moderating roles of these factors in the pathway of teachers’ readiness to teach and teacher motivation. Finally, the quantitative research methodology of cross-sectional data utilized in this study may not provide in-depth insight into the relationships between variables. Subsequent studies could use a mixed-research-method design to deeply characterize the relationships between variables.

Despite certain limitations, the theoretical and practical implications of this study cannot be ignored. First, this study pioneered the exploration of the link between teachers’ readiness to teach and the teacher–child relationship, and then we combined teachers’ motivation and self-efficacy to construct a mediational model that influenced the quality of the teacher–child relationship. This theoretical construct further enriches the diversity of research on proximal factors affecting the quality of teacher–child relationships. Second, this study emphasizes the pivotal role of teachers’ readiness to teach in fostering good teacher–child relationships. Therefore, policymakers, educational organizations, and teachers themselves must emphasize pre-service and post-service professional development and continuously enhance their professional knowledge and skills so that they can provide in-depth explanations and understanding of children’s characteristics and psychological needs, thus carrying out high-quality interactions with children [20]. Third, this study emphasizes the mediating roles of teachers’ motivation to teach and self-efficacy. This calls for teacher educators and preschool administrators to focus on cultivating teachers’ motivation (especially internal motivation) and self-efficacy levels in their professional development programs for teachers. Therefore, in the pre-service training courses and post-service training courses for kindergarten teachers, it is necessary to emphasize such training contents as teachers’ professional attitudes and values, along with career and vocational planning, to help teachers clarify their educational philosophy and core values and, thus, stimulate their intrinsic motivation for the career of kindergarten teachers. At the same time, it is also necessary to improve the working environment and treatment of kindergarten teachers, and to give them specific, positive, and timely feedback on their results and efforts, so as to stimulate their enthusiasm and motivation for their work. In addition, in pre-service and post-service training programs, relevant responsible entities should do a good job of researching professional development needs and designing teaching, to make up for the shortcomings of teachers’ knowledge and skills in a targeted manner, thus laying the groundwork for enhancing teachers’ self-efficacy. In summary, this study contributes to the understanding of the factors affecting teacher–child relationships and suggests a new development path to foster more positive teacher–child relationships in preschools. Thus, prioritizing and ensuring readiness to teach, along with enhancing the mediating roles of teaching motivation and self-efficacy, is essential for laying a strong foundation for effective and supportive learning experiences in early childhood.

## Figures and Tables

**Table 1:** Descriptive statistics and correlation results.

	1	2	3	4	5
1. Teaching readiness	1.00				
2. External motivation to teach	0.417 **	1.00			
3. Internal motivation to teach	0.346 **	0.635 **	1.00		
4. Teacher–child relationship	0.271 **	0.168 **	0.257 **	1.00	
5. Self-efficacy	0.262 **	0.247 **	0.244 **	0.305 **	1.00
*M*	3.13	3.60	3.80	3.59	3.99
SD	0.57	0.55	0.38	0.56	0.63

Note. ** *p* < 0.01.

**Table 2:** Regression results of variables in the models.

Regression Equation		Overall Fitting Index			Significance of Regression Coefficients	
Outcome Variables	Predictive Variables	*R*	*R^2^*	*F*	*β*	*t*
Model 1						
IMT	TR	0.35	0.12	39.07 ***	0.35	6.25 ***
SE	TR	0.31	0.10	15.03 ***	0.20	3.36 ***
	IMT				0.17	2.91 **
TCR	TR	0.39	0.15	16.85 ***	0.16	2.72 **
	IMT				0.15	2.47 *
	SE				0.23	3.96 ***
Model 2						
EMT	TR	0.42	0.17	60.33 ***	0.42	7.77 ***
SE	TR	0.30	0.09	14.38 ***	0.19	3.10 **
	EMT				0.17	2.70 **
TCR	TR	0.37	0.13	14.56 ***	0.20	3.17 **
	EMT				0.03	0.42
	SE				0.25	4.28 ***

Note: *** *p* < 0.001; ** *p* < 0.01; * *p* < 0.05. TR = teaching readiness; TCR = teacher–child relationship; IMT = internal motivation to teach; EMT = external motivation to teach; SE = self-efficacy.

**Table 3:** Standardized total, direct, and indirect effects, along with 95% confidence intervals.

Pathway	Estimate	Ratio	95% Confidence Interval	
			Lower	Upper
Model 1				
TR—IMT—TCR	0.051	18.82%	0.014	0.090
TR—IMT—SE—TCR	0.013	4.80%	0.004	0.028
TR—SE—TCR	0.046	16.97%	0.013	0.088
Total indirect effect	0.110	40.59%	0.058	0.172
Direct effect	0.161	59.41%	0.045	0.278
Total effect	0.271	100.00%	0.159	0.383
Model 2				
TR—EMT—TCR	0.011	4.06%	−0.041	0.058
TR—EMT—SE—TCR	0.017	6.274%	0.006	0.034
TR—SE—TCR	0.047	17.34%	0.013	0.094
Total indirect effect	0.075	27.68%	0.013	0.144
Direct effect	0.196	72.32%	0.074	0.317
Total effect	0.271	100.00%	0.159	0.383

Note: TR = teaching readiness; TCR = teacher–child relationship; IMT = internal motivation to teach; EMT = external motivation to teach; SE = self-efficacy.

## Data Availability

The data presented in this study are available on request from the corresponding author. The data are not publicly available due to ethical requirements.
